# Erythritol Availability in Bovine, Murine and Human Models Highlights a Potential Role for the Host Aldose Reductase during *Brucella* Infection

**DOI:** 10.3389/fmicb.2017.01088

**Published:** 2017-06-13

**Authors:** Thibault Barbier, Arnaud Machelart, Amaia Zúñiga-Ripa, Hubert Plovier, Charlotte Hougardy, Elodie Lobet, Kevin Willemart, Eric Muraille, Xavier De Bolle, Emile Van Schaftingen, Ignacio Moriyón, Jean-Jacques Letesson

**Affiliations:** ^1^Research Unit in Biology of Microorganisms, Department of Veterinary Medicine, University of NamurNamur, Belgium; ^2^Departamento de Microbiología y Parasitología, Instituto de Salud Tropical, Instituto de Investigación Sanitaria de Navarra, Universidad de NavarraPamplona, Spain; ^3^Laboratoire de Parasitologie, Faculté de Médecine, Université Libre de BruxellesBrussels, Belgium; ^4^WELBIO and de Duve Institute, Université Catholique de LouvainBrussels, Belgium

**Keywords:** *Brucella*, erythritol, aldose reductase, murine model, bovine trophoblast, human trophoblast, pentose phosphate cycle, polyol pathway

## Abstract

Erythritol is the preferential carbon source for most brucellae, a group of facultative intracellular bacteria that cause a worldwide zoonosis. Since this polyol is abundant in genital organs of ruminants and swine, it is widely accepted that erythritol accounts at least in part for the characteristic genital tropism of brucellae. Nevertheless, proof of erythritol availability and essentiality during *Brucella* intracellular multiplication has remained elusive. To investigate this relationship, we compared Δ*eryH* (erythritol-sensitive and thus predicted to be attenuated if erythritol is present), Δ*eryA* (erythritol-tolerant but showing reduced growth if erythritol is a crucial nutrient) and wild type *B. abortus* in various infection models. This reporting system indicated that erythritol was available but not required for *B. abortus* multiplication in bovine trophoblasts. However, mice and humans have been considered to lack erythritol, and we found that it was available but not required for *B. abortus* multiplication in human and murine trophoblastic and macrophage-like cells, and in mouse spleen and conceptus (fetus, placenta and envelopes). Using this animal model, we found that *B. abortus* infected cells and tissues contained aldose reductase, an enzyme that can account for the production of erythritol from pentose cycle precursors.

## Introduction

To survive and multiply any pathogen must harvest nutrients and consequently adapt to the carbon, energy and nitrogen sources that are available in the host. Pathogenesis is therefore not only a matter of virulence determinants, metabolism also enables virulence (de Lorenzo, 2014; Nataro, 2015). In this context, a paradigm of the correlation between metabolism and pathogenicity has been the preferential use of erythritol by the brucellae (McCullough and Beal, 1951; Smith et al., 1962), a group of facultative intracellular bacteria that cause brucellosis, a worldwide extended zoonosis (Zinsstag et al., 2011). A relevant part of the symptomatology of this disease is related to the particular tropism of the pathogen for the reproductive tract of ungulates and swine, which results in orchitis, epididymitis, abortion and infertility (Moreno and Moriyón, 2006). Massive intratrophoblastic colonization occurs in brucellosis by *B. abortus* in cows, *B. melitensis* in goats and sheep, *B. ovis* in sheep, *B. suis* in sows and *B. canis* in bitches (reviewed in Anderson et al., 1986b), and the infection of trophoblasts is a key step in the loss of integrity of the placenta that leads to abortion (Samartino and Enright, 1993) and subsequent dissemination. These events are critical in the biology of *Brucella* as these bacteria do not survive long in the environment and are transmitted mostly by contact with aborted tissues and fluids as well as venereally and congenitally (Moreno and Moriyón, 2006). Although not detected in early studies, more recent literature from endemic areas report a correlation between adverse pregnancy outcomes and *Brucella* infection (Khan et al., 2001; Karcaaltincaba et al., 2010; Al-Tawfiq and Memish, 2013; Vilchez et al., 2015), and epididymo-orchitis occurs in up to 20% of infected males (Navarro-Martinez et al., 2001).

The reasons for the preferential colonization of reproductive organs by the brucellae are not fully understood, and they may involve nutritional, immune, and hormonal factors (Samartino and Enright, 1993; Letesson et al., 2017). One of the molecular bases that is proposed to account, at least partially, for this tropism is the existence of erythritol in the target organs of ungulates (Smith et al., 1962). Present in substantial amounts in fetal fluids, placenta, seminal vesicles and semen of several ungulate species (Smith et al., 1962; Keppie et al., 1965; Clark et al., 1967), this four carbon polyol promotes *Brucella* growth at low concentrations and is also a preferred carbon source (McCullough and Beal, 1951; Smith et al., 1962). Bovine fetal tissues that were obtained from 6 to 7 months pregnant cattle (the time after which *Brucella* abortion often occurs) (Williams et al., 1962) and chorioallantoic membrane explants (Enright and Samartino, 1994) have been described to produce high amounts of erythritol. Nevertheless, other observations are not consistent with erythritol being the only factor in *Brucella* localization *in vivo.* First, although *B. ovis* and *B. canis* are unable to catabolize erythritol, they cause genital infections and abortion in sheep and dogs, respectively (Blasco, 1990; Carmichael, 1990). Second, vaccine *B. abortus* S19 is inhibited by erythritol (Jones et al., 1965) (see also below) and can cause genital infections and abortion in cattle. Third, high erythritol concentrations are not found in human or rodents (Keppie et al., 1965), hosts in whose reproductive tracts *Brucella* can localize and even multiply in cognate trophoblastic cell lines (Bosseray, 1980, 1983; Tobias et al., 1993; Kim et al., 2005; Salcedo et al., 2013). This last evidence, however, may need to be reinterpreted because *B. suis* mutants *eryB* and *eryC* (see below), which cannot catabolize erythritol, are attenuated in human macrophage-like THP-1 cells, murine J774 cells and BALB/C mice (Köhler et al., 2002; Burkhardt et al., 2005). As discussed below, the phenotype of these mutants suggests the presence of erythritol in these cells in amounts that are high enough to effect on *Brucella* multiplication.

**FIGURE 1:**
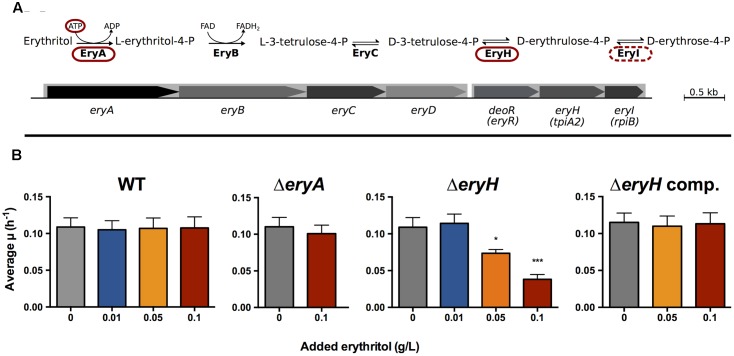
*Brucella abortus* Δ*eryA* and Δ*eryH* are erythritol-tolerant and erythritol-sensitive. **(A)** Revised erythritol catabolic pathway (the mutants used in this investigation are circled); **(B)** Growth of *B. abortus* 2308 WT, Δ*eryA*, Δ*eryH* and complemented Δ*eryH* mutants in 2YT medium supplemented with increasing concentrations of erythritol. Values are the average of biological and technical triplicates plus the standard deviation (^∗^*p* < 0.05; ^∗∗∗^*p* < 0.001 [Student’s *t*-test]).

The attenuation observed for some *ery* mutants has been attributed to the strong growth inhibition caused by erythritol that can be observed *in vitro* (Burkhardt et al., 2005). This bacteriostatic effect was identified very early as one of the markers of vaccine *B. abortus* S19 (Jones et al., 1965) and it was traced to an interrupted assimilation pathway (see below) (Sperry and Robertson, 1975). The mechanism of erythritol catabolism has been recently revised (Barbier et al., 2014) and proceeds through a five step pathway (**Figure 1A**). The first step, catalyzed by the EryA kinase, is an ATP-dependent phosphorylation that, while highly efficient, becomes futile when the downstream pathway is interrupted (i.e., when either EryB, EryC, EryH or EryI are not functional), as occurs in *B. abortus* S19. The consequence is that the ATP that was invested cannot be recovered and becomes depleted, hence the observed growth inhibition (Sperry and Robertson, 1975). This “toxicity” phenotype has prevented the carrying out of mutant-based analyses to investigate whether erythritol is actually used during infection and to what extent it contributes to *Brucella* multiplication during infection.

To clarify these issues, we exploited the phenotypes caused by mutations in different steps of the erythritol pathway as a reporting system. This system was based on a comparison of *B. abortus* 2308 wild type (WT) and mutants *eryA* (erythritol kinase) and *eryH* (isomerase; blocked in D-3-tetrulose-4-P/D-erythrulose-4-P conversion) (**Figure 1A**). As discussed above, the erythritol kinase mutant is erythritol-tolerant but, since it cannot metabolize erythritol, it should be attenuated if erythritol is a crucially needed nutrient (i.e., a major or even the only C source) in the replicative niche. However, the *eryH* mutant is predicted to be erythritol-sensitive, and therefore, it should be attenuated if erythritol is present. Using this reporting system, we found that erythritol was available but not required for *B. abortus* multiplication in bovine trophoblastic cells and, notably, also in human trophoblastic cells, in murine and human macrophage-like cells and in the spleen and conceptus of mice. These results led us to hypothesize that there should be a source of erythritol in tissues of mammals other than ungulates, and we present evidence for the involvement of the host aldose reductase (AR), an enzyme in the polyol pathway that can catalyze the synthesis of a plethora of polyols including erythritol.

## Results

### *B. abortus* 2308 *ΔeryA* and *ΔeryH* Mutants Are Erythritol-Tolerant and Sensitive, Respectively

We first constructed the non-polar mutants Δ*eryA* and Δ*eryH* of *B. abortus* 2308. Using a chemically defined medium, we confirmed for Δ*eryH* (Barbier et al., 2014) and demonstrated for Δ*eryA* their inability to grow with erythritol as the sole carbon source (**Supplementary Figure S1**). We found no growth defect in a rich medium that lacked erythritol (2YT [10% yeast extract, 1% tryptone, 5% NaCl]) (Dozot et al., 2010), a result that makes broader metabolic defects in these mutants unlikely. We made the same observation using a Δ*eryI* mutant (not shown), which was as expected.

A critical requirement for using these mutants as reporters of erythritol availability was that Δ*eryA* and Δ*eryH* should be erythritol-tolerant and erythritol-sensitive, respectively. To prove these correlation, we grew Δ*eryA* and Δ*eryH* in 2YT medium that was supplemented with increasing concentrations of erythritol (**Figure 1B**). Whereas erythritol did not affect the growth of the mutant Δ*eryA* at the highest concentration tested, it markedly inhibited the growth of the mutant Δ*eryH* at a concentration as low as 0.05 g/L, which is in the inhibitory range for vaccine S19 (Keppie et al., 1967) and for an *eryC* mutant of *B. suis* 1330 (Burkhardt et al., 2005). Since we could restore the WT phenotype of the Δ*eryH* mutant by complementation (**Figure 1B**), we concluded that deletion of *eryH* caused both the inability to grow on erythritol and its toxicity. As expected from the activity of EryI (downstream of *E*ryA, **Figure 1A**), the Δ*eryI* mutant was also inhibited by erythritol (not shown) and, as it phenocopied Δ*eryH*, below we present only the data obtained with the latter mutant.

Another critical requirement of any reporter system is specificity. To test the specificity, we studied the growth of WT and Δ*eryH* in 2YT medium supplemented with polyols (0.1 g/L) with structures close to erythritol (**Supplementary Figure S2**) that have been reported in male or pregnant female genital organs (Clark et al., 1967; Brusati et al., 2005; Jauniaux, 2005; Regnault et al., 2010; Larose et al., 2012). Since we did not detect any inhibitory effects for glycerol, ribitol, arabitol, xylitol, sorbitol, mannitol or dulcitol, the toxicity was specific for erythritol.

### Erythritol Is Available But Not Essential for *B. abortus* Multiplication in Bovine and Human Trophoblastic Cells

**FIGURE 2:**
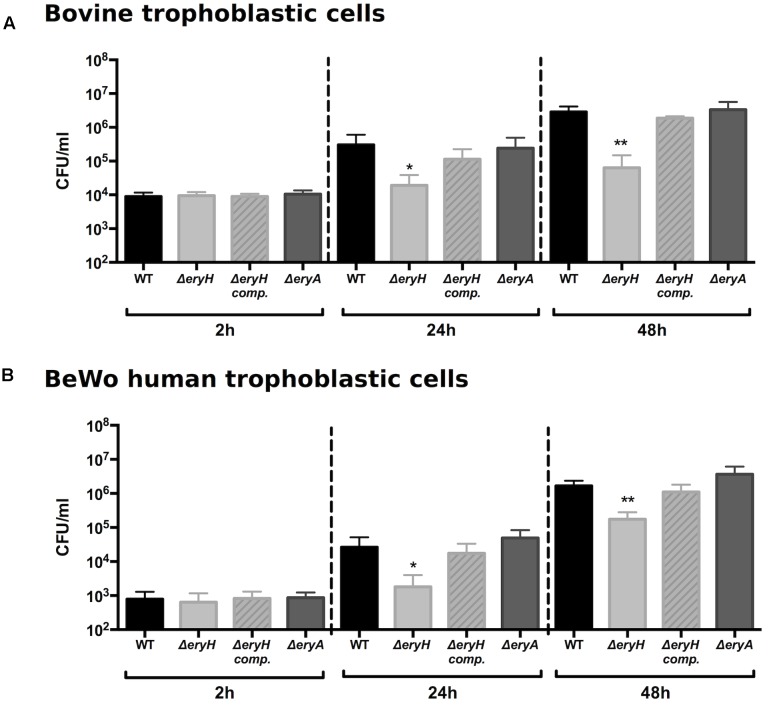
*Brucella abortus* Δ*eryH* but not *B. abortus* 2308 Δ*eryA* or the complemented *B. abortus* Δ*eryH* (*eryH* comp.) are attenuated in bovine **(A)** and human trophoblastic **(B)** cells. Values are the average of biological and technical triplicates plus the standard deviation (^∗^*p* < 0.05; ^∗∗^*p* < 0.01; ^∗∗∗^*p* < 0.001 [Student’s *t*-test]).

Since brucellae multiply intensively in bovine trophoblasts (Anderson and Cheville, 1986; Anderson et al., 1986a), we tested our reporting system in a suitable bovine trophoblastic cell line (Samartino et al., 1994). As shown in **Figure 2A**, no differences could be evidenced between the WT and the mutants at 2 h post infection (p.i.), a time when bacteria have not yet reached their replicative niche (Samartino et al., 1994). At later times, Δ*eryH* (erythritol-sensitive) but not Δ*eryA* (erythritol-tolerant) failed to multiply at the level of the WT strain. These results are consistent with the availability of erythritol in the replicative niche of *B. abortus* in bovine trophoblasts.

These observations and the recent description that *Brucella* can colonize human trophoblastic cell lines (Salcedo et al., 2013; Fernandez et al., 2016) prompted us to test our reporting system in trophoblastic cells other than those of bovine origin. When we infected human BeWo trophoblastic cells with *B. abortus* 2308 WT, Δ*eryA* or Δ*eryH*, we observed that the mutants were indistinguishable from the WT 2 h after infection and that only the multiplication of the erythritol-sensitive mutant Δ*eryH* was significantly reduced at later times (**Figure 2B**). These observations are in apparent conflict with previous studies that reported only very low erythritol concentrations in human fetal tissues (Keppie et al., 1965; Clark et al., 1967; Amin and Wilsmore, 1997). However, results obtained with cell lines do not necessarily reflect the *in vivo* situation and the parallelism with the results in bovine trophoblastic cells indicated that, while not being required for optimal bacterial replication, erythritol should be available in human BeWo cells at a concentration above that which is toxic for the sensitive mutant.

### Erythritol Is Available But Not Essential for *B. abortus* Multiplication in RAW 264.7 and THP-1 Macrophage-Like Cells

**FIGURE 3:**
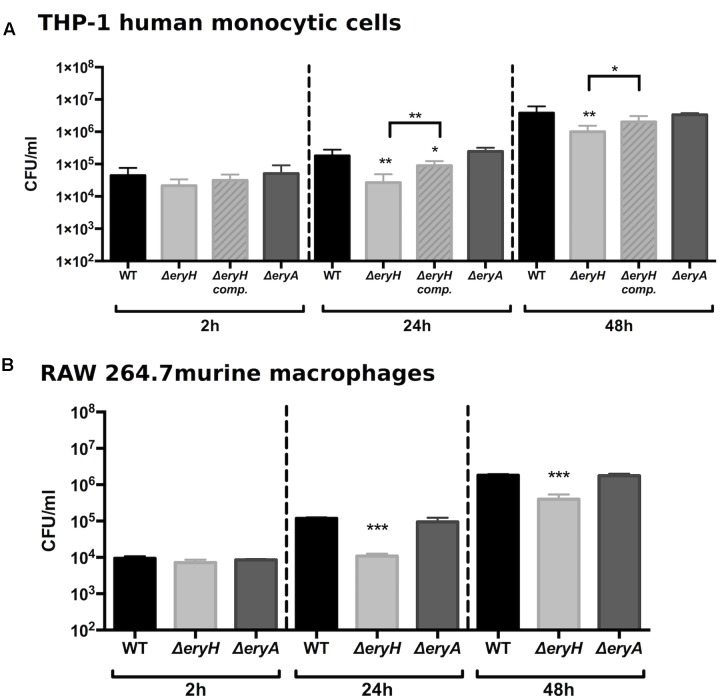
*Brucella abortus* Δ*eryH* but not *B. abortus* 2308 Δ*eryA* or the complemented *B. abortus* Δ*eryH* (*eryH* comp.) are attenuated in human **(A)** and murine **(B)** macrophage-like cells. Values are the average plus the standard deviation of three experiments for THP-1 and of one representative replicate for RAW 264.7 (^∗^*p* < 0.05; ^∗∗^*p* < 0.01; ^∗∗∗^*p* < 0.001 [Student’s *t*-test]).

Macrophages are another cell type in which *Brucella* multiplies extensively. For this reason, RAW 264.7 murine macrophages and THP-1 human macrophage-like cells were infected with the erythritol catabolic mutants (**Figure 3**). Although both were able to invade these cells to the same extent as the WT (i.e., resulted in the same CFU/ml at 2 h p.i.), the erythritol-sensitive mutant was found in lower numbers 24 and 48 h after infection. These results are in agreement with those of Burkhardt et al. (2005) who showed that a *B. suis* 1330 Δ*eryC* mutant was erythritol-sensitive and attenuated in macrophages. Indeed, these authors also reported Δ*eryC* attenuation in mice and suggested an availability of erythritol *in vivo*.

### Erythritol Availability and Essentiality Are Time-Dependent in C57BL/6 Mouse Spleens

**FIGURE 4:**
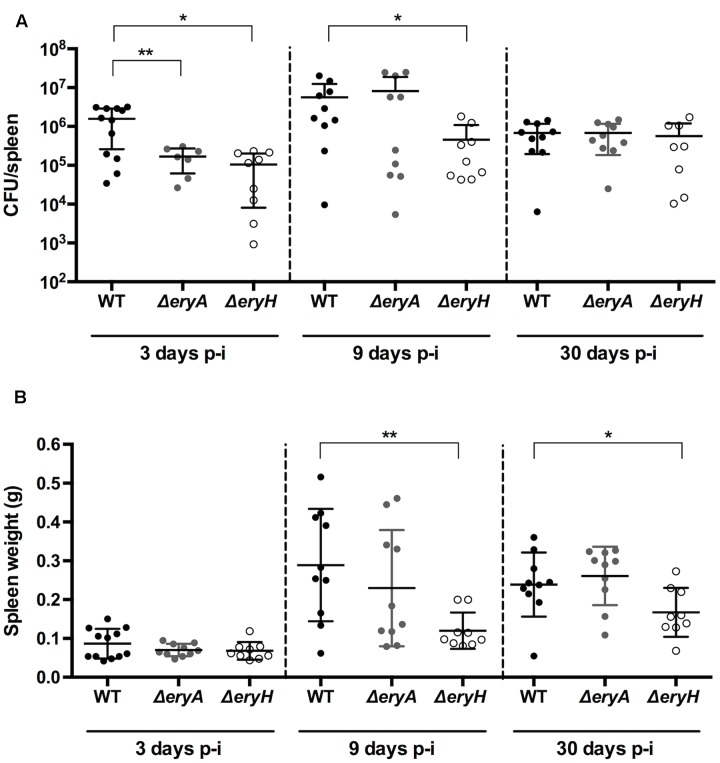
The *B. abortus* erythritol catabolic mutants Δ*eryA* and Δ*eryH* are attenuated in C57BL/6 mice. Mice were infected with 5 × 10^4^ CFU, and CFU in the spleen **(A)** and spleen weights **(B)** determined at the indicated intervals. Each dot represents the CFU counts of one individual mouse, and bars the mean ± standard deviation (^∗^*p* < 0.01; ^∗∗^*p* < 0.001; Mann–Whitney test).

In C57BL/6 mice that were infected intraperitoneally with either *B. abortus* 2308 WT or the erythritol catabolic mutants, the CFU numbers/spleen of both Δ*eryA* and Δ*eryH* were significantly lower than those of the WT 3 days after infection (**Figure 4**). After 9 days, only the erythritol-sensitive mutant showed significantly reduced CFU numbers, and consistent with this, splenomegaly was lower in the corresponding group of mice. Although splenomegaly was reduced in the Δ*eryH-*infected group, no differences in the CFU numbers of the mutants were observed 30 days after infection. These results are also in line with those of Burkhardt et al. (2005) who also found reduced CFU/spleen for the *B. suis* Δ*eryC* at early times (7 days or less) but not 28 days after infection. On the other hand, they are only in partial agreement with those of Sangari et al. (1998). These authors found that the CFU/spleen of a *B. abortus* 2308 erythritol sensitive Tn5 mutant and the parental strain were similar 7, 14, and 28 days after infection. The discrepancy is thus limited to the results obtained 7 days after infection but as earlier times were not studied and the CFU/spleen at longer times coincide with the our observations, it seems possible that the differences could relate to the protocols (breed of mice [BalB/c versus C57BL/6], Tn5 polarity effects on regulators downstream and possibly others).

Bearing in mind the properties of the mutants in our reporting system, these results suggest that the availability and importance of erythritol as a carbon source in the spleen of mice changes during the course of infection. During a splenic infection everything including the bacterial load, the type of infected cells, the type of recruited cells, the splenic microarchitecture and the immune environment is evolving in a dynamic way. Therefore, it may be not so surprising that 3 days after infection (that is to say before the peak of splenic infection and before any detectable splenomegaly) *Brucella* is in a compartment where erythritol is available (attenuation of Δ*eryH*) but also required (attenuation of Δ*eryA*); later on, changes in one or several parameters (i.e., infected cells or immune environment) might lead to a change in the nutrients that are available which makes erythritol, while available (attenuation of Δ*eryH*) less needed (no attenuation of Δ*eryA*). Afterward, erythritol availability would progressively dwindle because both mutants reached a WT-like bacterial load at 30 days p.i.

### Erythritol Is also Available in the *B. abortus*-Infected Murine Conceptus

**FIGURE 5:**
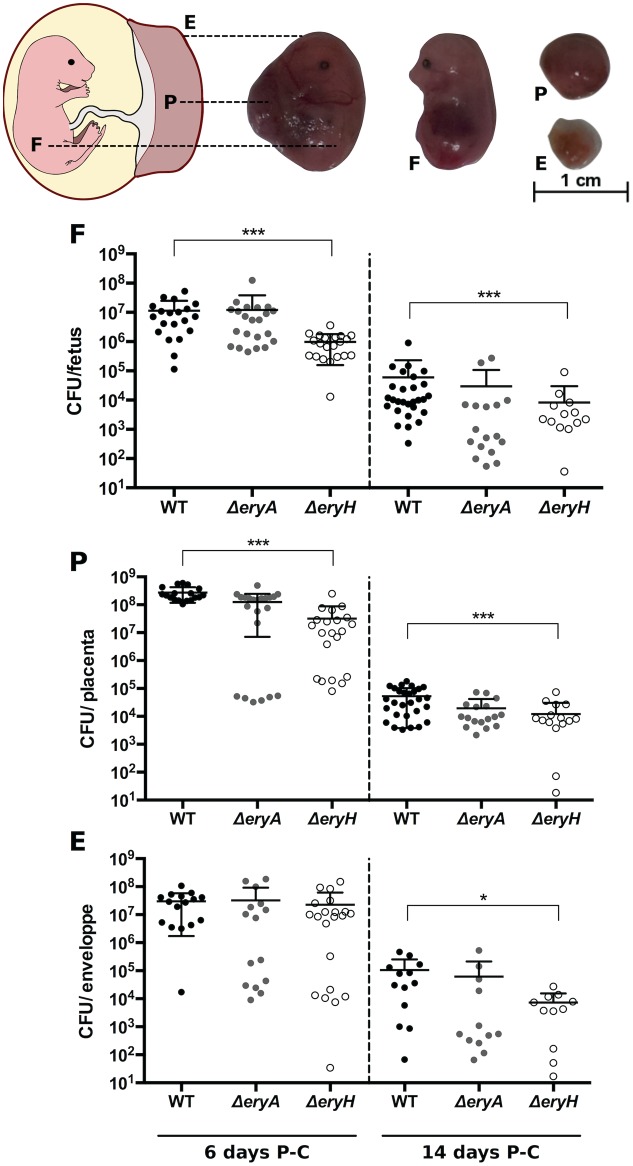
*Brucella abortus*Δ*eryH* but not Δ*eryA* show reduced ability to colonize the mouse conceptus. Pregnant C56BL/6 mice were inoculated intraperitoneally with 10^5^ *B. abortus* 2308 WT, Δ*eryH* or Δ*eryA* mutant at 6 or 14 days post conception (P-C). At day 15 P-C, individual conceptuses were dissected to separate the placenta (P), the fetus (F) and the fetal envelopes (E) and CFU determined. Each dot represents the CFU counts obtained from one conceptus and bars represent the mean ± standard deviation (^∗∗^*p* < 0.01; ^∗∗∗^*p* < 0.001; Mann–Whitney test).

The well-known genital tropism of *Brucella* prompted us to investigate erythritol availability in the mouse conceptus. We infected C57BL/6 mice in either early (6 days) or late (14 days) pregnancy with *B. abortus* WT, Δ*eryA* and Δ*eryH* (Bosseray, 1980). Then, we determined the CFU in fetuses, placenta and fetal envelopes 1 and 9 days p.i., which in both groups corresponds to post-conception day 15. The results (**Figure 5**) showed a systemic distribution of the three strains in the conceptus with the placenta being be the most heavily infected organ, as expected from its barrier function (Bosseray, 1980). For the mice that were infected at day 6 post-conception, only the erythritol-sensitive strain Δ*eryH* was present in significantly lower numbers in the fetus and placenta. When mice were infected late in pregnancy, only Δ*eryH* was attenuated, and attenuation was observed in all tissues. These results strongly suggest that erythritol is available but not crucially required for *B. abortus* to multiply in the murine conceptus.

### Aldose Reductase Is Expressed in RAW 264.7 Macrophages and Tissues Infected by *B. abortus*

The polyol pathway has been suggested to be involved in the synthesis of erythritol in mammalian tissues (Pearce et al., 1962) and the key enzyme, AR, can catalyze the conversion of erythrose into erythritol (Hayman and Kinoshita, 1965; Kardon et al., 2008). Thus, we examined whether there was a connection between AR and the ability of *Brucella* to multiply in cells and tissues.

**FIGURE 6:**
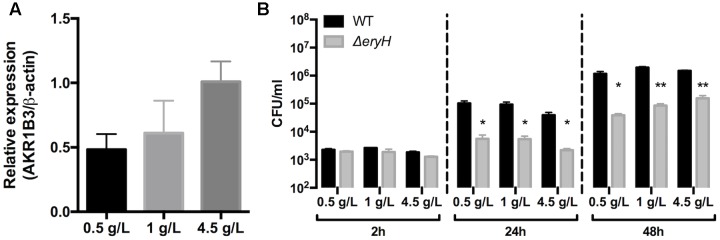
The expression of Aldose reductase gene Akr1b3 in RAW 264.7 macrophages depends on glucose concentration. **(A)** Expression of gene Akr1b3 measured by qRTPCR in macrophages cultured with 0.5, 1, and 4.5 g/L of glucose. **(B)** Multiplication of *B. abortus* 2308 WT and Δ*eryH* in macrophages cultured with 0.5, 1, and 4.5 g/L of glucose. All experiments were performed in biological and technical duplicates (^∗^*p* < 0.05; ^∗∗^*p* < 0.01; ^∗∗∗^*p* < 0.001 [Student’s *t*-test]).

First, we studied whether AR is expressed in some of the cells in which our reporting system indicated the presence of erythritol. We found that the enzyme was detectable by immunofluorescence and that AR gene expression was observed in uninfected RAW 264.7 macrophages that were cultured in standard media; no noticeable changes were noted when these cells were infected by *Brucella* (data not shown). Since AR is induced by hyperglycemia (≥20 mM glucose; 5 mM being normoglycemic) (González et al., 1984; Tawata et al., 1992) and the DMEM-HG culture medium contains 25 mM glucose, we also measured the dependence of the expression of gene Akr1b3 (which codes for mouse AR) on glucose as an indirect and complementary test for AR activity. In RAW 264.7 macrophages that were grown in a range (2.8 [0.5 g/L], 5.6 [1.0 g/L] and 25 mM [4.5 g/L]) of glucose concentrations, we found that expression of Akr1b3 was modulated by glucose (**Figure 6A**), a result that strongly suggests that AR is active in these cells in culture. Although the glucose concentration did not affect the multiplication of the Δ*eryH* mutant during the first 2 h after infection, its inhibition was significant at 24 and 48 h (**Figure 6B**) suggesting that erythritol is available in cells that are grown in the range of physiological glucose concentrations that induce AR gene expression.

**FIGURE 7:**
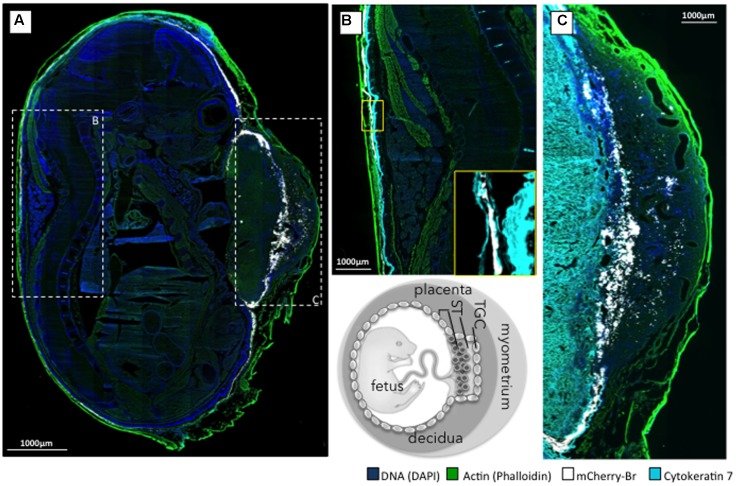
Localization of *B. abortus* 2308 in murine conceptus. Pregnant mice were infected intraperitoneally at 6 days post-conception with 10^5^ CFU of *B. abortus* mCherry (white), and the conceptuses were obtained at post-conception day 15. **(A)** Mosaic reconstitution (210 individual images taken at 10× magnification) of a sagittal section of an infected murine conceptus stained for DNA (DAPI/blue) and actin (phalloidin/green); dashed squares B and C circumscribe the prototypal zone corresponding to **(B,C)**. **(B)** Cytokeratine-7 immunostaining (light blue) of trophoblastic cells of the dorsal part of the conceptus with a close up of the infected trophoblastic cells lining the decidua parietalis (yellow square). **(C)** Cytokeratine-7 immunostaining (light blue) of the placenta at the level of the decidua basalis. TGC, trophoblast giant cell; ST, spongiotrophoblast; L, labyrinth.

**FIGURE 8:**
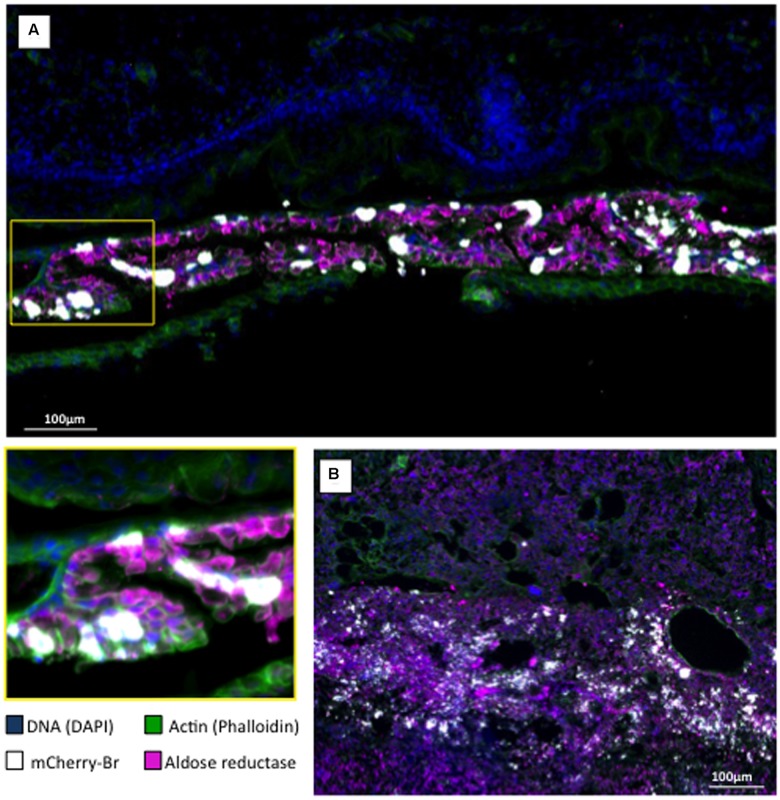
Aldose reductase localization in *B. abortus* infected murine conceptus. Pregnant mice were intraperitoneally infected at post-conception day 6 with 10^5^ CFU of a *B. abortus* mCherry (mCherry-Br; white color). Conceptus were obtained at post-conception day 15 and stained for DNA (DAPI/blue) and actin (phalloidin/green). **(A)** Aldose reductase immunostaining (purple) of the trophoblastic layer surrounding the fetus (the area in the yellow square corresponds to the lower left panel of the Figure). **(B)** Aldose reductase immunostaining (purple) of the placenta at the level of the decidua basalis.

Second, we examined the AR and bacterial distributions in the conceptuses of mice that were infected intraperitoneally with *B. abortus* 2308-mCherry at 6 days post conception. Bacteria and AR-positive cells were located preferentially in the junctional zone of the placenta just underneath the decidua basalis and in a cellular sheet surrounding the fetus apposed on the internal face of the distended decidua parietalis (**Figure 7A**). Infected cells were positive for cytokeratin 7 (**Figures 7B,C**) and thus trophoblastic in nature (Croy et al., 2013), probably being trophoblastic giant cells (Kim et al., 2005). As expected, some infected trophoblasts were also scattered in the decidua after mid-gestation (Hu and Cross, 2010). Bacteria were found almost exclusively in AR-positive cells (**Figure 8**).

Finally, we investigated mouse spleens 9 days after infection, a time at which the erythritol-sensitive strain Δ*eryH* showed attenuation. We found intense AR-staining in the red pulp (**Supplementary Figure S3**), with small clusters of CD11b-positive cells [often iNOS positive and corresponding to granulomas (Copin et al., 2012)], which are frequently associated with AR. In contrast, we hardly detected AR in spleens of non-infected mice; when we did, AR was mostly restricted to scattered CD11b-negative cells in the red pulp.

## Discussion

In this work, we set up and validated a reporting system to detect the presence and catabolism of erythritol in the *Brucella* replicative niche, and this system demonstrated the availability of this polyol in infection models of bovine, human and murine origin, extending previous research in macrophages and mice (Burkhardt et al., 2005) to trophoblastic cell lines. Indeed, because erythritol is present in comparatively large amounts in the placenta and genital tissues of ruminants and swine and because *Brucella* is found inside trophoblastic cells of ruminants and uses erythritol very efficiently, it has been widely assumed that trophoblasts produce erythritol. However, most evidence is limited to extracts of fetal allantoic and amniotic fluids, cotyledons, whole placenta, seminal vesicles and testis (Smith et al., 1962; Williams et al., 1962; Clark et al., 1967), and to the best of our knowledge, only one work has reported the presence of erythritol in trophoblasts of bovine origin (Enright and Samartino, 1994). Our work confirms this pattern and provides the first experimental data that support the presence of erythritol in human and murine trophoblastic cells. In addition, we demonstrate for the first time that the catabolism of erythritol is not essential to the infectious processes in these infection models.

The fact that bovine and human trophoblastic cells and murine models give similar results contrasts with the low erythritol concentrations that were reported in fetal fluids of humans and mice (approximately 60 μg/ml in cows and less than 2 μg/ml in humans or mice) (Keppie et al., 1965; Amin and Wilsmore, 1997). Based on our *in vitro* toxicity assays in 2YT, it can be speculated that erythritol concentration should reach 50–100 μg/ml to result in Δ*eryH* attenuation during infection. If correct, these differences in erythritol measurements in fetal fluids could reflect the particular composition of the *Brucella* replicative niche during infection. It is apparently puzzling that, although our reporting system shows that erythritol is catabolized “*in vivo*,” it also shows that erythritol is not an essential carbon source, indicating a complex nutritional situation in the replicative niche where erythritol, but also alternative C sources, should be available in various and evolving proportions based on the time of infection, the type of cell infected and other variables. A possible explanation for this situation could be the presence of an active polyol pathway because this pathway (which depends on AR; see below) can supply not only erythritol but also other polyols such as glycerol, arabitol, mannitol and inositol that are in fact found in fetal tissues and reproductive systems of several *Brucella* hosts (Jauniaux, 2005). These polyols, erythritol included, may not be critical individually but could be alternative carbon sources. Of course, a definite answer needs specific investigations carried out in the natural host species, not only with *B. abortus*, *B. melitensis* and *B. suis* but also with *B. ovis* and *B. canis*, the two classical species not stimulated by erythritol.

Since our reporting system showed erythritol in cells in which its presence has not been described, we looked for possible biosynthetic mechanisms in mammal tissues. Over 50 years ago, Pearce et al. (1962) proposed that erythritol “*may arise from D-erythrose [ …] as an intermediate between D-erythrose and D-erythrulose as sorbitol acts as an intermediate between glucose and fructose*.” The enzyme responsible for the conversion of glucose to sorbitol is the aldose reductase (AR, AKR1B1 in human and bovines, AKR1B3 in mice) of the polyol pathway, which oxidizes sorbitol to fructose. Thus, we investigated the presence of AR in cells and tissues where *B. abortus* multiplied and where the presence of erythritol was detected. We found that AR was present in RAW 264.7 macrophages independently of an infection, that 9 days after infection there was a sharp increase in AR in the splenic red pulp where clusters of CD11b^+^ (indicative of *Brucella*-induced granulomas) cells co-localized with AR and that AR and *B. abortus* co-localized in the infected murine conceptus. There is abundant indirect evidence that this coexistence of AR and brucellae in the laboratory models parallels the situation in the natural hosts. Actually, tissues characteristically targeted by brucellae such as the placenta of cows, sheep and pigs, and the epididymis, seminal fluids and oviduct of pigs, cattle and some rodents, which are among those tissues whose fructose concentrations are high or are predominant over glucose, contain abundant amount of AR (Clark et al., 1967; Frenette, 2006; Pruneda et al., 2006). Moreover, AR (AKR1B1) has also been recently identified by proteomics as differentially produced in bovine chorioallantoic membranes that were infected by *Brucella* (Mol et al., 2016). Indeed, AR can reduce a broad range of aldehydes to their corresponding alcohols (Håstein and Velle, 1968) and significantly its affinity is far higher for erythrose than for glucose (Hayman and Kinoshita, 1965; Kardon et al., 2008). Furthermore, a role of AR in erythritol generation in these tissues is consistent with the fact that the pentose phosphate pathway, which can supply D-erythrose, is active in testes, ovaries and placenta (Ferrier, 2013). Although further research is necessary, all these indirect evidences together with the data presented here, lend support to the hypothesis that AR accounts for erythritol production in cells that have been invaded by brucellae, as well as for the apparently puzzling observation that erythritol is not essential for *Brucella* multiplication. In preliminary experiments, we have found that treatment of murine macrophages with the potent AR inhibitor Sulindac (Ratliff et al., 1999) impairs *B. abortus* 2308 intracellular multiplication, an observation that is also consistent with our hypothesis. It is also worth commenting that AR is a moonlighting protein that in addition to its function in the polyol pathway, has been linked to inflammation regulation (Ramana and Srivastava, 2010) and is involved in the hormonal regulation of pregnancy and parturition. Some AR are, as a matter of fact, involved in progesterone degradation and are also the main human, murine and bovine prostaglandin F2α (PGF2α) synthase (Madore et al., 2003; Kabututu et al., 2008; Bresson et al., 2011, 2012). It is thus tempting to hypothesize that AR could represent an actor in the context of *Brucella*–host interaction at the crossroad of metabolism, inflammation and abortion, a possibility that deserves further investigation.

## Materials and Methods

### Bacterial Strains and Culture Conditions

*Escherichia coli* DH10B were grown in LB medium. *B. abortus* 2308 Nal^R^ and derived strains were grown at 37°C in rich medium 2YT (16 g/L bacto tryptone 10 g/L yeast extract and 5 g/L NaCl; BD Difco) or in a chemically defined medium (Barbier et al., 2014) composed of 2.3 g/L K_2_HPO_4_; 3 g/L KH_2_PO_4_; 0.1 g/L Na_2_S_2_O_3_; 5 g/L NaCl; 0.2 g/L nicotinic acid; 0.2 g/L thiamine; 0.07 g/L pantothenic acid; 0.5 g/L (NH_4_)_2_SO_4_; 0.01 g/L MgSO_4_; 0.1 mg/L MnSO_4_; 0.1 mg/L FeSO_4_; 0.1 mg/L biotin and 2 g/L of erythritol. Growth was monitored using an automated plate reader (Bioscreen C, Lab Systems) following the OD (600 nm) with continuous shaking at 37°C. The growth rate was calculated as follows: (In (ODt2) − In (ODt1)) / (t2 − t1). The Δt was set for 7 h, i.e., approximately two division times, and incremented over the log phase every 0.5 h (0–7 h; 0.5–7.5 h,…) resulting in a set of values whose mean is the average growth rate, μ. When required, the medium was supplemented with chloramphenicol (20 mg/ml), nalidixic acid (25 mg/ml), sucrose (5%), agar (15 g/L, BD Difco) and polyols (concentrations annotated in the manuscript). Unless otherwise stated, reagents were purchased from Sigma–Aldrich.

Construction of an mCherry-producing *B. abortus* 2308 strain was performed following the same procedure that was validated for *B. melitensis* (Copin et al., 2012). Construction of the in-frame deletion in *eryA* was done following a previously described strategy (Barbier et al., 2014). Briefly, approximately 750 bp upstream and downstream of BAB2_0372 were amplified by PCR from genomic DNA of *B. abortus* 2308. The obtained PCR products were, respectively, flanked by SpeI/BamHI (SpeI_F: 5′-ACTAGTCTTGGCGGAAACTTGACTGG-3′; BamHI_R: 5′-ATACGCGGATCCGCGATAACGCATGGCTGACACAGG-3′) and BamHI/SphI restriction sites (BamHI_F: 5′-TATCGCGGATCCGCGTATGGCAAATAAGGAAACATTGAATG-3′; SphI_R: 5′-GCATGCGCGCTTGTCGTGGTTCTG-3′). A third PCR joined the two fragments together using primers SpeI_F and SphI_R, which was followed by ligating this product into an EcoRV-digested pGEM plasmid (Promega). After sequence verification (Beckman Coulter Genomics), the ±1500 bp insert was excised as a SpeI – SphI fragment and cloned into a pNPTS138 suicide vector (Kan^R^, Suc^S^). The acquisition of this vector by *Brucella* after mating with conjugative S17 *E. coli* was selected by kanamycin and nalidixic acid resistance. The loss of the plasmid concomitant with either a deletion or a return to WT phenotype was then selected on sucrose. Mutants were identified using PCR with primers that were located external to the deletion. The Δ*eryH* and Δ*eryI* strains were previously characterized (Barbier et al., 2014). For complementation, *eryH* was amplified by PCR from genomic DNA of *B. abortus* 2308 as a BamHI/XhoI fragment (BamHI_F: 5′-gcgggatccatgaccaaattctggattgg-3′; XhoI_R: 5′-ttaattcgcttgaaccttggctcgagccg-3′). Fragments were cloned into an EcoRV-digested pGEM, sequenced and then transferred into a pBBR1MCS1 (Cm^R^). The Δ*eryH* strain with the construct was then transformed by conjugation with the construction and selected for with the newly acquired resistance to chloramphenicol.

All *Brucella* were handled under BSL-3 containment according to the Directive 98/81/CE du Conseil du 26 octobre 1998 and to a law of the Gouvernement wallon du 4 juillet 2002.

### Cell Culture and Infection

RAW 264.7 murine macrophages were routinely cultured in Dulbecco’s modified Eagle’s medium with high glucose (DMEM, Gibco) supplemented with 10% heat-inactivated fetal calf serum (FCS, Gibco). THP-1 human macrophage-like cells were cultured in RPMI 1640 medium (Gibco) that was supplemented with 10% FCS and 2 mM L-glutamine. Cells were differentiated into adherent monocytes by overnight treatment with 5 nM phorbol myristate (PMA). Bovine trophoblastic CL2 cells were kindly provided by Pr. Cynthia Baldwin (University of Massachusetts, Amherst, MA, United States) and cultured in RPMI 1640 supplemented with 10% FCS and 0.05 mM 2-mercaptoethanol (Gibco) as previously described (Parent et al., 2012). BeWo human trophoblastic cells (ATCC clone CCL-98) were cultured in DMEM-F12 Ham medium (Gibco) that was supplemented with 10% FCS and 2 mM L-glutamine as already described (Salcedo et al., 2013). All cells were maintained at 37°C with a 5% CO_2_ atmosphere.

For experiments in which RAW 264.7 were cultured with various glucose concentrations, DMEM with no glucose (Gibco) was supplemented with the appropriate amount of filter-sterilized glucose. Cells were cultured under these conditions for at least 2 passages prior to infection. Infections were performed as described elsewhere (Salcedo et al., 2013; Zúñiga-Ripa et al., 2014). Briefly, cells were seeded in 24-well plates at an appropriate density (2.105 cells/ml THP-1 cells and 4.104 cells/ml for RAW 264.7 and trophoblastic cells) and infected 24 h later with a multiplicity of infection (MOI) of 100. The cells were centrifuged at 1000 RPM for 10 min at 4°C before being incubated for 1 h at 37°C with 5% CO_2_; they were then washed with fresh medium and incubated for 1 h with medium containing 50 μg/ml of gentamicin. The medium was then replaced with a fresh medium that contains 10 μg/ml of this antibiotic. At 2, 24, and 48 h after infection, cells were washed with PBS and treated for 10 min at room temperature with PBS Triton X100 0.1%; after this treatment, the lysates were collected, diluted, plated on TSB and incubated at 37°C for approximately 3 days to enumerate the CFUs.

For immunofluorescence staining, cells were seeded on coverslip and treated as described previously (Francis et al., 2017). At the end of the process, the cells were fixed in 2% paraformaldehyde, pH 7.4, at 37°C for 15 min.

### C57BL/6 Mice Infection

The procedures used and the handling of mice complied with current European legislation (directive 86/609/EEC) and the corresponding Belgian law “Arrêté royal relatif à la protection des animaux d’expérience du 6 avril 2010 publié le 14 mai 2010.” The Animal Welfare Committee of the Université de Namur (Belgium) reviewed and approved the complete protocols (Permit Number 16/277). All infections were performed at an Animal Biosafety Level 3 facility.

To obtain the inoculum, bacteria from an overnight culture of *Brucella* in rich medium were pelleted, washed with RPMI 1640 and diluted in this medium. Intraperitoneal infection was carried out as previously described (Copin et al., 2012). Briefly, 500 μl of suspension (10^5^ CFU) was injected into groups of 8 to 12 C57BL/6 mice for each tested strain. Mice were euthanized 3, 9, and 30 days post-infection by cervical dislocation, the spleens were isolated, weighted and homogenized in 1 ml of PBS Triton X100 0.1%, and the CFU were counted on tryptic soy agar plates.

The procedure that was used to infect pregnant mice was adapted from previous reports (Bosseray, 1982; Kim et al., 2005; Pennington et al., 2012). Estruses of 6–14 weeks old C57BL/6 females were synchronized 3 days before mating pairs were set up with males that were 3–4 months old. Then, the presence of a vaginal plug was checked daily, and potentially fertilized females were isolated. That day corresponds to day 0 post-fecundation (PF). Four to five pregnant females were infected intraperitoneally with 500 μl of bacterial suspension that was prepared as previously described (10^5^ bacteria) at day 6 or 14 PF. At day 15 PF, mice were anesthetized with isoflurane (Zoetis) and euthanized by cervical dislocation. Conceptuses were removed from maternal uterine horns and transferred to sterile Petri dishes on ice, where they stayed for 15 min. Placenta, fetuses and surrounding fetal membranes were then further isolated and weighed. Tissues were homogenized in 1 ml PBS Triton X100 0.1% with an Ultra-Turrax homogenizer, the homogenates were serially diluted in PBS, and their CFU were counted.

### Immunofluorescence Microscopy

Fetuses and spleens were fixed for 2 h at room temperature in 2% paraformaldehyde (pH 7.4), washed in PBS, and incubated overnight at 4°C in a 20% PBS-sucrose solution. The tissues were then embedded in Tissue-Tek OCT compound (Sakura) and frozen in liquid nitrogen, and cryostat sections (thickness, 5 μm for spleens and 10 μm for fetus) were prepared. For the staining, tissue sections were rehydrated in PBS and incubated in a PBS solution that contained 1% blocking reagent (PBS-BR 1%, Boehringer) for 20 min before they were incubated overnight in PBS-BR 1% containing mAbs or the following reagents: DAPI nucleic acid stain Alexa Fluor 350 or 488 phalloidin (Molecular Probes) to visualize the structure of the organ, and rat biotin-coupled anti-mouse CD11b (BD Pharmingen), rabbit anti-mouse iNOS (Calbiochem), rabbit anti-mouse Cytokeratin 7 (ab 181598, Abcam), and rabbit anti-mouse AR (*CPA3124*, Cohesion Biosciences) to stain the cells of interest. The samples were incubated with the appropriate secondary reagents [Alexa Fluor 568 streptavidin (Molecular Probes) or Alexa Fluor 647-coupled donkey anti-rabbit IgG (Molecular Probes)] for 2 h. Slides were mounted in Fluoro-Gel medium (Electron Microscopy Sciences, Hatfield, PA, United States). Labeled tissue sections were visualized with an Axiovert M200 inverted microscope (Zeiss, Iena, Germany) that was equipped with a high-resolution monochrome camera (AxioCam HR, Zeiss).

Images (1384 pixels × 1036 pixels, 0.16 μm/pixel) were acquired sequentially for each fluorochrome with A-Plan 10×/0.25 N.A. and LD-Plan-NeoFluar 63×/0.75 N.A. dry objectives and recorded as eight-bit gray-level ^∗^.zvi files. At least three slides per organ were analyzed from three different animals, and the results are representative of two independent experiments.

For immunostaining of AR in RAW 264.7 murine macrophages, the primary antibody that was used was the same that was used for staining in mice with a goat anti-rabbit IgG Alexa 488 (Life Technologies) as the secondary antibody.

### Measurement of the Murine Aldose Reductase AKR1B3 Expression in RAW 264.7 by qRT-PCR

RNA from cells cultivated in a T75 flask was extracted with TriPure isolation reagent (Roche) according to the instructions of the manufacturer and DNA contamination was eliminated by incubation with DNase I (Fermentas). Then, RNA was first reverse transcribed (two steps) by SuperScript II (Invitrogen) into cDNA, which was then amplified in a LightCycler 96 Instrument (Roche) with FastStart Universal SYBR Green Master (Roche) as the fluorescent dye. The specificity of the SYBR Green assays was assessed by melting-point analysis and gel electrophoresis. The results were normalized using the housekeeping b-actin gene. Primer sequences are described in Supplementary Table S1.

## Author Contributions

TB, AZ-R, IM and J-JL conceived the study. AM and EM were responsible for the immunofluorescence microscopy analysis. XDB supervised all the molecular approaches. HP, CH, and EL contributed in the mutant construction, growth curves and tested them in cells and mice. TB and AZ-R were the main researchers involved in mutant and metabolic tests. EVS brought a lot of input in the aldose reductase and polyol pathway. J-JL, TB, and IM wrote the paper. All the authors read and commented on the paper.

## Conflict of Interest Statement

The authors declare that the research was conducted in the absence of any commercial or financial relationships that could be construed as a potential conflict of interest.

## Supplementary Material

**FIGURE S1 |***Brucella abortus* 2308 Δ*eryA* and Δ*eryH* are not able to grow with erythritol as the only carbon source. The growth of the Δ*eryA* mutant was monitored in rich medium 2YT or in chemically defined medium with erythritol as the only carbon source and compared to Δ*eryH* {Barbier:2014 cm}. As expected, the growth of the deletion strains is abolished when only erythritol is available.

**FIGURE S2 |** The growth of the erythritol-sensitive *B. abortus* 2308 Δ*eryH* strain is not affected by other polyols. Growth was monitored in 2YT supplemented with polyols structurally close to erythritol that are also found in fetal fluids and tissues. The growth of the Δ*eryH* mutant was not affected by any of the polyols tested strongly suggesting that the toxicity is specific for erythritol. The experiment was performed in biological triplicates and technical duplicates. Values represent the average of one representative experiment ± standard deviation (shaded gray and dashed lines).

**FIGURE S3 |** Aldose reductase localization in the spleen of mice infected with *B. abortus* 2308. Mice were infected intraperitoneally with 10^5^ CFU of *B. abortus* 2308, spleens obtained at day 9 post infection and stained for actin (phalloidin/gray) and CD11b (green). **(A)** iNOS immunostaining (blue) of spleen sections of non-infected (control, Left), and infected (Br.9d, Right), mice. The red pulp shows small clusters of CD11b-positive cells (often iNOS positive) that correspond to *Brucella* granulomas {Copin:2012ee}. **(B)** Aldose reductase immunostaining (purple) of spleen sections of non-infected and infected mice. In the non-infected mice (control, Left), aldose reductase was scarcely detected and was restricted to a few and CD11b-negative cells of the red pulp. In infected mice 9 days post-infection (Br.9d, Right), aldose reductase was abundant in the red pulp in the same areas where the *Brucella* granulomas developed.
