# Supplementary figures and images for: Erythritol Availability in Bovine, Murine and Human Models Highlights a Potential Role for the Host Aldose Reductase during Brucella Infection
Source: Front Microbiol. 2017 Jun 13;8:1088. doi: 10.3389/fmicb.2017.01088 (PMC5468441; doi:10.3389/fmicb.2017.01088)

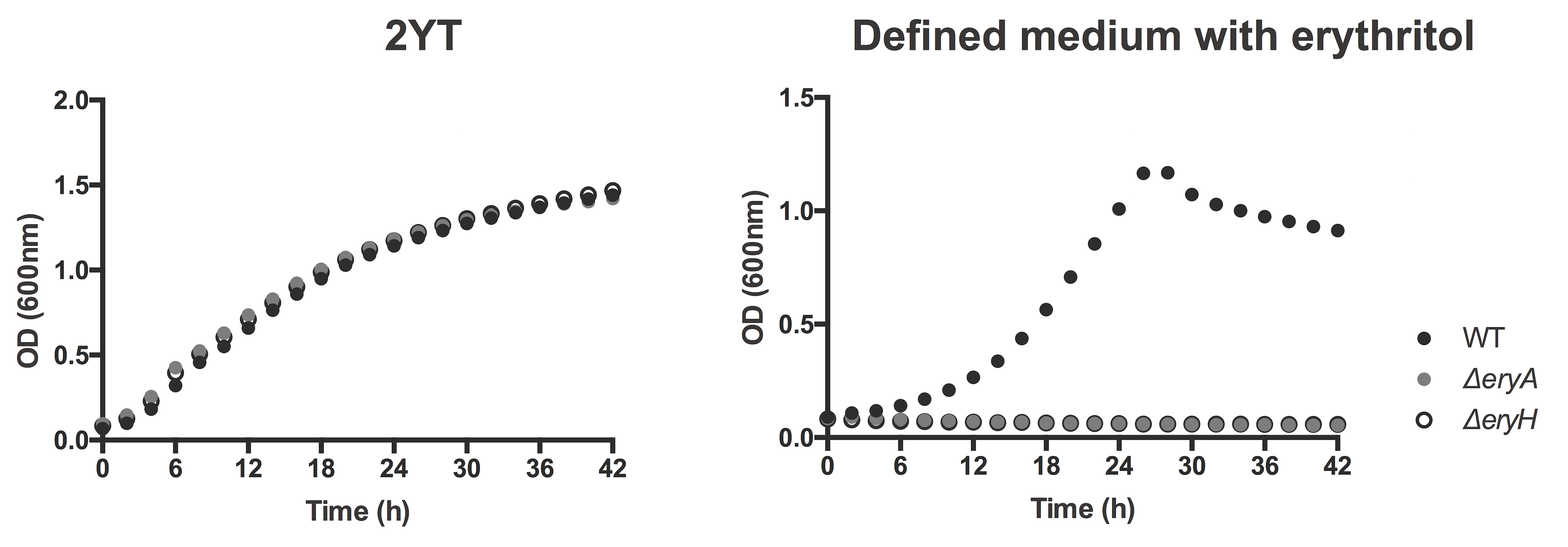

Supplement: Supplementary file 1 [file Image_1.TIFF]

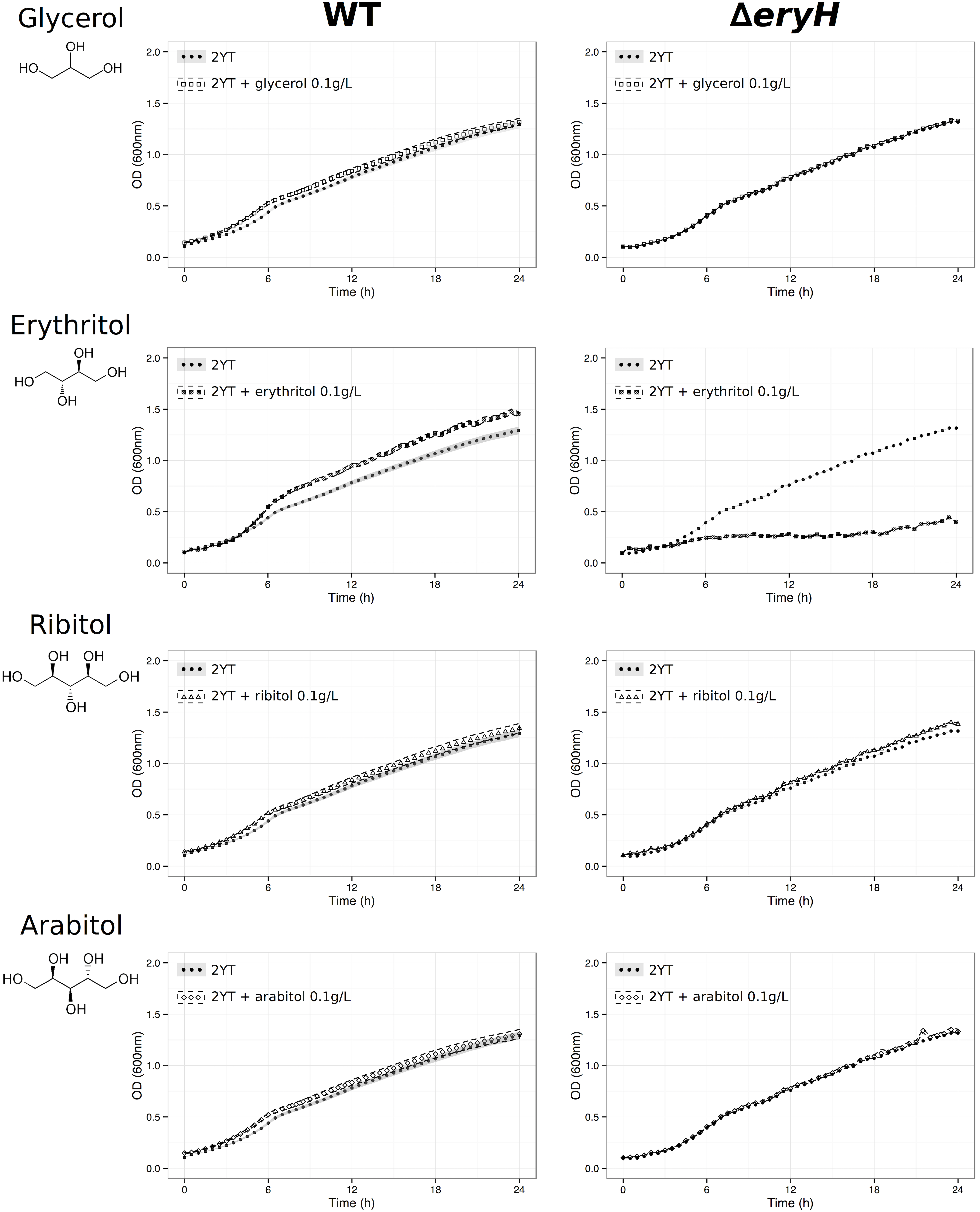

Supplement: Supplementary file 2 [file Image_2.TIFF]

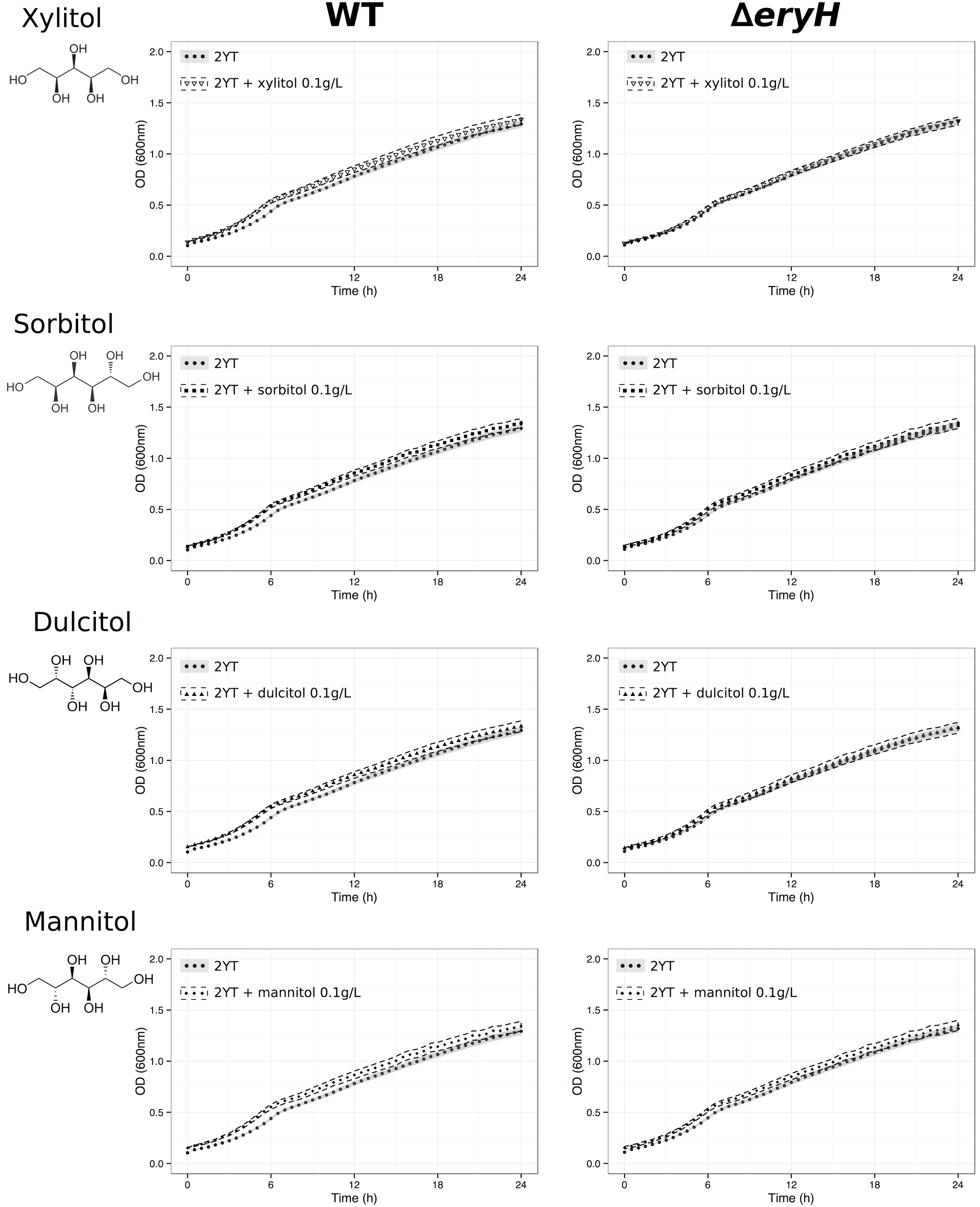

Supplement: Supplementary file 3 [file Image_3.TIFF]

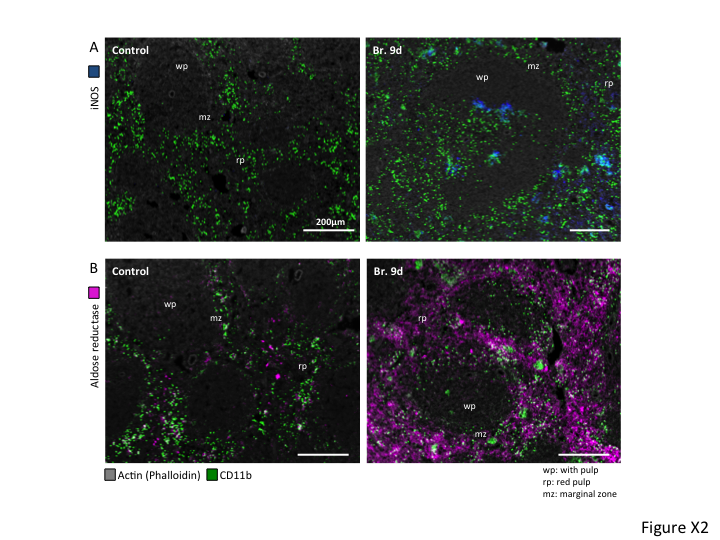

Supplement: Supplementary file 4 [file Image_4.TIFF]
