# Supplementary material for: Erythritol Availability in Bovine, Murine and Human Models Highlights a Potential Role for the Host Aldose Reductase during Brucella Infection
Source: Front Microbiol. 2017 Jun 13;8:1088. doi: 10.3389/fmicb.2017.01088 (PMC5468441; doi:10.3389/fmicb.2017.01088)
Supplement: Supplementary file 5 [file Table_1.docx]

Table S1 primers used

| **Name of the primer** | **Sequence** |
| --- | --- |
| Forward AKR1B3 (mouse) | ctggatgcctcagggaacgtgatacc |
| Reverse AKR1B3 (mouse) | agagggttgaagttggagacaccgattg |
| Forward B actin (mouse) | acggccaggtcatcactattgg |
| Reverse B actin (mouse) | gtttcatggatgccacaggattcc |
